# Outcomes of a Parent-Delivered Baby-mCIMT Model for Infants at High Risk of Unilateral Cerebral Palsy Using Remote Coaching in Telerehabilitation

**DOI:** 10.3390/children11010101

**Published:** 2024-01-15

**Authors:** Katarina Svensson, Heléne Sundelin, Ann-Christin Eliasson

**Affiliations:** 1 Division of Children’s and Women’s Health, Department of Biomedical and Clinical Sciences, Linkoping University, 58183 Linkoping, Sweden; katarina.svensson@liu.se; 2 Crown Princess Victoria’s Children Hospital, 58185 Linkoping, Sweden; 3 Neuropediatric Unit, Department of Women’s and Children’s Health, Karolinska Institute, 17177 Stockholm, Sweden; helene.sundelin@ki.se; 4 Neuropaediatric Research Unit, Astrid Lindgren Children’s Hospital, 17176 Stockholm, Sweden

**Keywords:** infant, unilateral CP, constraint-induced movement therapy, early intervention, telerehabilitation, internet-based training, hand function, hand assessment for infants

## Abstract

There is growing evidence of the positive effects of constraint-induced movement therapy (CIMT) for infants at high risk of unilateral cerebral palsy (UCP) when provided by parents with in-person coaching/supervision from occupational therapists during home visits. The aim of this study is to investigate whether Baby-mCIMT (modifiedCIMT) can be as effective if parents are coached/supervised remotely. In this case-control study, we recruited 20 infants and re-used 18 controls, 4–8-month-old infants in both groups at high risk of UCP. The same protocol regarding inclusion criteria, data collection, and training volume was used in both groups. The training was conducted for two 6-week periods, separated by a 6-week break, consisting of daily 30 min sessions conducted by parents, supported by therapist coaching once a week. The primary outcome was measured using the Hand Assessment for Infants (HAI). There was no difference in the change of HAI units (*p* = 0.803) or that of the affected-hand raw score (*p* = 0.942) between the two groups. The remote coaching method was well received by parents. In conclusion, this demonstrates that remote coaching/supervision is as effective as the in-person approach, requiring less time and effort for both families and healthcare providers.

## 1. Introduction

There is growing evidence suggesting that early intervention improves motor skills in infants at high risk of cerebral palsy (CP) [1,2,3]. This is particularly relevant to the training of upper limbs in infants at high risk of unilateral cerebral palsy (UCP) [3,4,5,6]. Constraint-induced movement therapy (CIMT) is the most common method for intensive intervention addressing the upper extremities, but there is no established “gold standard” for organizing and implementing such training to achieve optimal results. Most studies in children use a modified version of the signature CIMT model [7]. Parents, supported by professional interveners, have often been the ones to conduct this training in various modified CIMT programs for infants [4,8], but other models, such as camps and hospital-based programs, are also available [9,10,11]. However, all CIMT variants are time-consuming for both families and healthcare providers and, therefore, probably not cost-effective. With an increased experience of and improved opportunities for digital communication brought about by the COVID-19 pandemic [12,13,14], it is of interest to investigate whether Baby-mCIMT provided by parents can be more efficient and produce the same results using telerehabilitation, i.e., digital communication in coaching and supervision.

Telerehabilitation has several advantages, such as accessibility, time efficiency, and cost-effectiveness, for both families and healthcare providers, as the communication between them occurs digitally and remotely [15,16]. There are few well-controlled studies of telerehabilitation for children with CP, most lacking breadth and methodological quality [17]. However, several studies report positive results for the feasibility of telerehabilitation for children with various diagnoses [18,19]. Coaching is a key element of telerehabilitation, but there is no generally accepted definition of coaching, only various definitions ranging from relationship-directed to intervener-directed concepts without a clear distinction between the instructing and coaching of parents [19,20,21]. Coaching is generally found to be a viable intervention in early intervention studies because the model of parent coaching is strongly aligned with the scope of professional interveners [21] and has commonly also been used in other recent early intervention studies [5,22,23]. Nevertheless, there is a need for further investigations of telerehabilitation, as few studies compare its treatment effects with those of established and evidence-based in-person methods [17,18,19]. The most effective telerehabilitation programs appear to incorporate coaching methods and parental implementation [19]. We, therefore, assumed that telerehabilitation using digital remote coaching/supervision might have the potential to improve hand function in infants with effects similar to those of the in-person approach supported by home visits.

In this study, our previously reported randomized control trial (RCT) of a Baby-mCIMT program conducted by parents in the home environment, coached and supervised by occupational therapists during home visits [5,24], has been further developed for remote coaching/supervision (i.e., remote Baby-mCIMT). Both programs have the same underlying concepts and training intensity, but in remote Baby-mCIMT, the home visits are replaced with video meetings, chat communication, and a patient-adapted web platform. We chose to call the earlier approach in-person Baby-mCIMT in this study. Our hypothesis is that remote Baby-mCIMT has the same effect on the children’s hand function as in-person Baby-mCIMT since human support is the most important component of both online and in-person coaching [25]. To our knowledge, this study is the first to compare remote versus in-person coaching for a parent-delivered Baby-mCIMT model for infants at high risk of UCP using HAI as the outcome measure.

## 2. Materials and Methods

### 2.1. Design

This case-control exploratory study was conducted between 2018 and 2022 at Habilitation and Health, Region Stockholm, which was responsible for the intervention as part of its clinical assignment. The control group consisted of infants who had undergone Baby-mCIMT in a previous RCT [5]. The study was conducted in accordance with the Declaration of Helsinki and approved by the Institutional Review Board (or Ethics Committee) of the Stockholm Regional Ethical Review Board (no. 2015/4:9, 2018/1900-32, 2009/1100-32).

### 2.2. Participants

The remote Baby-mCIMT group consisted of 20 infants, and the retrospective control group consisted of 18 infants, for a total of 38 participants. The infants in both groups were recruited from various clinics and follow-up programs at Karolinska University Hospital, except for two infants recruited from the Southeastern Region of Sweden. To be eligible, potential participants had to be infants 3–8 months of corrected age (CA) with a ≥15% difference between their two hands as assessed using the Hand Assessment for Infants (HAI) [26]. The participants also had to be considered at high risk of developing UCP based on a known neonatal event affecting the brain and/or clinical signs identified using assessments such as the Hammersmith Infant Neurological Examination (HINE) [27]. The exclusion criteria were (1) severe visual impairment, (2) seizures that could not be controlled by antiepileptic drugs, and (3) families who could not communicate in either English or Swedish. All parents received oral and written information before providing written informed consent. The perception of the intervention among the families was evaluated. An additional four families in the remote group were included in this evaluation, although they were excluded from the intervention outcome part of the study because their children did not meet the inclusion criterion for age.

### 2.3. Intervention

#### 2.3.1. Characteristics of Remote and In-Person Baby-mCIMT

The CIMT training in this study differs from the signature model developed by Taub and fits with the definition of modified CIMT [7,28]. It is defined as training of the affected hand when the non-involved hand is restricted, but in this case, by a mitten or something similar that is comfortable and well-accepted by the child. The infants in both groups underwent 30 min daily interventions, 6 days a week, for two 6-week periods separated by a 6-week break, for a total of 36 h [24], conducted by the parents in the infants’ home environment. Once a week, the training was conducted under supervision and coaching by an occupational therapist and, in two cases, by a physical therapist. The training included several components, of which grasping actions and toy exploration were the main focus, as described in more detail in the study protocol [24]. A specific training focus was specified each week depending on the infant’s ability and progress. The training focus was formulated and written down in collaboration with the parents before the sessions ended, and the actual duration of training was noted in a diary.

#### 2.3.2. Differences between Remote and In-Person Baby-mCIMT

The main difference between the intervention programs is that the therapist’s weekly home visits in the control group were replaced with digital meetings, a further adjustment of the mCIMT model. In the home-based in-person program, the therapist could explore the children’s abilities by testing different behaviors and toys themselves, while in the digital meetings, the parents were guided to do the same. The families in the remote Baby-mCIMT program had access to web-based instructions and information. In addition, in the in-person Baby-mCIMT group, one occupational therapist conducted all interventions, while six different clinical occupational therapists and one physiotherapist with no formal training other than access to the Baby-mCIMT manual conducted the interventions in the remote group. Nevertheless, each of them possessed a minimum of 15 years of experience in pediatrics.

#### 2.3.3. Specific Characteristics of the Remote Baby-mCIMT Program

The remote Baby-mCIMT program was administered through Habilitation and Health, an organization that provides support and assistance to children and adults with disabilities. The remote Baby-mCIMT program used the national e-health platform “Stöd och behandling” (https://www.inera.se/tjanster/alla-tjanster-a-o/stod-och-behandling, accesed on 1 December 2023), available in Sweden. This platform includes digital features such as video links, messaging, and logbook options and ensures high patient confidentiality and data security. Also included is supporting information about CIMT, such as videos, slideshows, and a library of pictures and videos of suitable toys. One home visit was recommended, mainly to strengthen the relationship with the families, but this was rarely done when COVID-19 precautions were in place.

During the weekly session, the infant had to be seated in front of a smartphone, tablet, or computer, whichever was used for the meeting. The session started with a short review of the past week. The parents were then asked to start the training by giving the infant toys commonly used during the training and playing in the usual way. The therapist observed, listened, and made suggestions, such as “What happens if you present the toy in a different way or if you change toys?” This was followed by a discussion on how to proceed and what would be the focus of the following week. This focus was noted in the web platform.

### 2.4. Procedure for Assessment

HAI was administered in the clinic before and after each training period, a total of four times in both groups during the study period. In the control group, the HAI assessment was scored by one blinded rater after the intervention period, while in the remote Baby-mCIMT group, HAI was scored by the different clinicians responsible for the intervention after each training period.

#### 2.4.1. Assessments of Outcome

HAI, a standardized observation-based test for infants 3–12 months old who are at risk of developing UCP [26], has good inter-rater reliability [29]. It assesses the degree and quality of goal-directed manual actions performed with each hand separately as well as both hands together. The test procedure consists of a semi-structured, video-recorded 10–15 min play session. A test kit of carefully selected toys is presented to the infant, making a wide range of motor actions observable. HAI contains 17 items (12 unimanual and five bimanual) scored on a three-point rating scale. The sum of raw scores is transformed using Rasch analysis into the “both-hands” measure on a 0–100 HAI-unit scale, with a higher score indicating better performance. For the unimanual items, each hand is scored separately, with a raw score range of 0–24 for each hand; additionally, the asymmetry index is calculated as the percentage difference between the unimanual raw scores [26].

The parents’ perception of the remote intervention was evaluated using a questionnaire with six predetermined questions, each with four response options, after the interventions; we evaluated the perceptions of the in-person Baby-mCIMT group using four of these questions.

#### 2.4.2. Baseline Measurement

The Alberta Infant Motor Scale (AIMS) tools, describing gross motor development relative to a norm-referenced sample [30], were used to establish the baseline. CP was diagnosed employing the criteria of the Surveillance for Cerebral Palsy in Europe (SCPE) (2000). Brain lesions were characterized based on available neuroimaging, collected for clinical purposes at various times using various protocols. The basic patterns of damage were classified as normal, white-matter damage of immaturity (WMDI), focal ischemic or hemorrhagic lesions, brain malformations, and miscellaneous or unclassifiable lesions [31].

### 2.5. Statistical Analysis

All analyses were conducted using the SPSS 29.0 software. Demographic data were described and examined for differences between groups (Table 1). Data were found to violate the assumption of normality, so non-parametric statistics were applied. Possible differences in results between intervention groups were measured using the Mann–Whitney U test. The Wilcoxon signed-rank test was used to compare the within-group change over time for each corresponding data point for both groups. Spearman rank correlation was used to explore the correlations between age (CA) at baseline and the change in HAI units after intervention and the correlation between HAI units at baseline and the change in HAI units after the intervention period. In all cases, the significance level was set at *p* < 0.05.

## 3. Result

### 3.1. Characteristics of the Study Population

All participants completed the training program, and there were no reported adverse events. The duration of the intervention was somewhat prolonged in the remote Baby-mCIMT group, i.e., a mean of 26 weeks compared with 23 weeks in the in-person Baby-mCIMT group (*p* = 0.026) due to a longer break between intervention periods. Gestational age was somewhat higher in the remote group, i.e., 38 (sd = 3.2) compared with 34 weeks (sd = 6.5) in the in-person group (*p* = 0.001), with more infants born at term in the remote group. No between-group difference in age (CA) was at the HAI baseline. Neuroimaging showed signs of lesions that could explain the CP in all assessed infants (no radiology, *n* = 6). At the end of the intervention, a high risk of CP was confirmed in all infants except for one in the remote Baby-mCIMT group. This infant had an asymmetry index of 19% at inclusion (at 6 months of age), but the final assessment at 11 months showed no asymmetry and a score of 100 HAI units. In the remote Baby-mCIMT group, two children did not meet the criteria for UCP at the age of 2 years [5]. Further details are found in Table 1.

### 3.2. Outcome in HAI Units and Affected Hand Raw Score after Intervention

**Figure 1:**
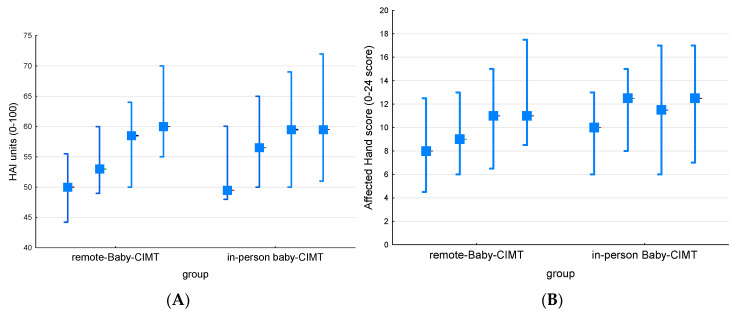
Median and interquartile range (IQR) at baseline, after the first intervention period, after a pause (i.e., before the second intervention period), and at the last assessment (four assessments for each intervention period) for the remote and in-person coaching Baby-mCIMT groups: (**A**) both-hands measure in HAI units (0–100) and (**B**) affected-hand raw score (0–24).

No significant differences were found between the two groups in terms of change in HAI units (*p* = 0.803) or in affected-hand raw score (*p* = 0.942, Figure 1 and Table 2). The HAI units increased in both groups (*p* = 0.001), as did the affected-hand raw score (remote group, *p* = 0.001; in-person group, *p* = 0.005).

### 3.3. Individual Variation and Correlations

**Figure 2:**
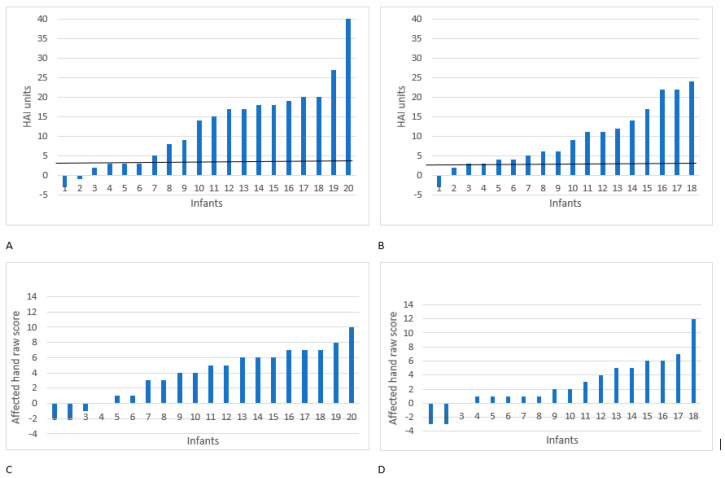
Individual variation in changed score from baseline to end of training in (**A**) HAI units after remote coaching Baby-mCIMT; (**B**) HAI units after in-person coaching Baby-mCIMT; (**C**) affected hand raw score after remote coaching Baby-mCIMT; (**D**) affected-hand raw score after in-person coaching Baby-mCIMT. Horizontal line is ≥3, the smallest detectable difference.

There were large intra-individual variations in HAI outcomes (Figure 2). However, most infants had an increase of ≥3 HAI units, which is the smallest detectable difference (SDD) for HAI [29], except for three in the remote group and two in the in-person group. Age (CA) at baseline displayed no significant correlation to HAI improvement after intervention in either the remote group (*r* = 0.314, *p* = 0.178) or the in-person group (*r* = 0.169, *p* = 0.501). The correlation between the severity (HAI units at baseline) and the improvement in terms of HAI units was also not significant in either the remote group (*r* = 0.113, *p* = 0.634) or the in-person group (*r* = 0.377, *p* = 0.123).

### 3.4. Outcome in HAI Units during Different Intervention Periods

Both groups improved after the first (remote Baby-mCIMT, *p* ≤ 0.002; in-person Baby-mCIMT, *p* ≤ 0.001) and second intervention periods (*p* = 0.001 and *p* = 0.048, respectively). Although the median value did not change in the second intervention period, there were significant improvements in both groups since the Wilcoxon signed rank test also considers the distribution and ranking of individual observations.

There seem to be improvements between treatments, although these are not significant (remote Baby-mCIMT, *p* = 0.079, in-person Baby-mCIMT, *p* = 0.517).

### 3.5. Feasibility of the Intervention

Most parents found it very easy to conduct the training at home (i.e., remote group 79%, in-person group 65%), although slightly more parents in the remote group found it difficult or very difficult (Table 3). Almost all parents (i.e., remote group 93%, in-person group 100%) found the support from the occupational therapist to be of great or some importance (Table 3). Although most families (i.e., remote group 93%, in-person group 100%) found the effect good or somewhat good, one family (i.e., 7%) in the remote group did not perceive any effect. This family also found it very difficult to perform the training. The information material seemed to be perceived as useful, with all families in the remote group reporting that it was very or fairly informative and inspiring. The web information was unavailable to two families that only participated in the remote meetings; one of them only spoke English. All but one family in the remote group would recommend the training to other families in a similar situation. Nine families’ answers to the questionnaire in the remote group are missing due to technical problems.

## 4. Discussion

In agreement with our hypothesis, the effect of the remote Baby-mCIMT was equal to that of the in-person Baby-mCIMT assessed in the earlier RCT. These results were influenced by neither age at baseline nor impairment severity. The individual variation in outcome was huge in both groups, as some infants displayed minor and some great improvements, in line with other studies [32,33,34].

Improvements were also observed, especially in the remote group during the intermission, although not significant. A possible explanation might be the delayed effects of treatment or the sustained impact of continuous bimanual stimulation, as also observed in our previous study [35]. This study demonstrates that the digital environment for supervising and coaching parents did not influence the effect of the intervention. It is also important to note that the remote model was already fully implemented in the clinic. It is still difficult to compare the results to those of other studies since there are only a few published studies of CIMT in the youngest age group. Three relevant studies were found in a recent systematic review, but all used different assessments of outcomes [4]. Recognizing the limitations of this design, we acknowledge that this study can be considered practice-based evidence at best.

### 4.1. Digital Coaching versus In-Person Coaching

Telerehabilitation incorporating digital coaching of parents has recently been shown to be effective [19]; however, the contents in terms of methods of coaching and supervision vary and are rarely well described. Graham et al.’s [20] general definition of coaching contains emotional support, information exchange, and a structured process that includes elements such as setting goals, exploring options, planning action, implementing plans, and checking performance. Traditional coaching for improved motor function typically involves the therapist explaining to families what to do since coaching is commonly used in combination with an exercise program [19]. The in-person Baby-mCIMT program was inspired by Graham et al. [20] and used a problem-solving coaching approach together with a motivational interview to promote knowledge transfer and capacity building in parents to increase their skills at improving infant functioning [36,37]. During home visits, the therapist typically plays with the child and acts as a role model in addition to using the coaching model. In contrast, the therapist in remote Baby-mCIMT in digital meetings must rely entirely on the coaching model to support the parents and, in various ways, provide productive and positive feedback on the families’ attempts to achieve progress. In remote coaching, non-verbal communication cues, including body language and gestures, are missing, and extended wording and various stylistic procedures and emoticons are needed to overcome this [25]. This difference might affect parents’ learning processes. We found the greatest improvement in the first period in the in-person group, while improvement appeared later in the program in the remote group. This might reflect the different coaching environments and perhaps even the length of the digital meeting, which usually did not last more than 30–40 min, while home visits lasted at least one hour. Our experience is, however, that both parents and therapists were more focused and better prepared during the digital meetings. For successful coaching, it is also important to build a good collaborative relationship between families and therapists [38,39,40]. We thought that such a relationship would be difficult to build in a digital meeting, which was the reason for suggesting one home visit in the remote model, although this was only partly possible during the period of COVID-19 precautions. A combined model of in-person and remote coaching has also been suggested and discussed in other coaching programs [25,41]. However, a recent study comparing an in-person with a virtual empowerment-focused parenting intervention program designed to promote family health in low-income countries identified good opportunities to build relationships and strengthen social networks via digital meetings [42]. This is in agreement with Santarossa et al. [25], who suggested that human support is the most important factor for both in-person and online coaching.

### 4.2. Feasibility of the Program

When evaluating different interventions, it is crucial to set criteria in advance for the successful feasibility of an intervention [43]. For home-based training, accessibility in terms of opportunity to perform the training, compliance, motivation, and technical ease are all important [44,45]. In this study, we concluded that both the accessibility and the training compliance were good in both groups since there were no dropouts and the families appreciated the support of the occupational therapists, both in person and digitally. Similar feasibility results have been reported for other kinds of home-based programs incorporating remote support, such as care-toy training and eTIPS [22,44]. This is in contrast to a program assessing gross motor function in which the adherence decreased to 83% [12]. No adverse events were reported for either of the groups studied here, in line with previous studies of CIMT programs [2] and of digital programs for other diagnoses or forms of training [44,46]. Parental motivation to conduct the training can, to some extent, be assessed from responses to questions about how the effect of training was perceived and whether it was fairly easy to perform the training. There was only a minor difference in the responses between the groups, indicating that the feasibility was good for both approaches. We found it difficult to assess whether the parents would prefer remote or in-person programs because of how the relevant question would be perceived and answered by families with infants, as most had no experience with the other alternative. Provenzi et al. (2021) reported that up to 40% of respondents rated a telehealth family-centered rehabilitation program for children with disabilities, administered during the COVID-19 lockdown, to be as effective as or more effective than usual care [46]. Most of these families reported increased feelings of engagement, self-relevance, perceived support, and recognition in their role as caretakers for their children [46]. We collected data from both the parents’ and therapists’ perspectives, which will be reported in a separate paper. Across both groups, all families, except for one in the remote group, would recommend the training to other families in similar situations.

### 4.3. Limitations

One limitation was that the strict procedure used in the RCT could not be followed in this study. Another significant limitation was the absence of blinded assessors in the remote group, potentially introducing bias into the assessment results. The recruitment procedure was different, as infants at high risk of UCP were referred to Habilitation and Health from Karolinska University Hospital, and these families could access the intervention even if they did not participate in the study. At the time of the RCT, there were no options to have the intervention other than participating in the study. In the remote group, the children had slightly greater impairment at baseline. On the other hand, this can also be seen as a strength since it is known that severely impaired children are less likely to improve over time [47]. There is also some improvement during the intermission, with even greater changes noted in the remote group. This is difficult to interpret; it is unclear whether this signifies a delayed effect of the training or if parents continued the training during the intermission. Although the parents were instructed to cease one-hand training, they were allowed to continue with general stimulation of bimanual hand use.

Another limitation was the lack of fidelity measures, as we only collected information on the frequency and duration of the intervention and nothing about adherence to therapy [48]. The families were asked to note training time in a log book, but these diary notifications went missing due to technical problems. According to information from the therapists, however, most families performed the 30 min of daily training, although some parents did not and a few families attended only a small number of digital meetings. The seven therapists in the remote group were experienced in pediatrics’ but were less experienced in CIMT. They received only a brief introduction before commencing remote supervision and coaching of the families, whereas one therapist employed in the research unit treated all children in the RCT. The Baby-mCIMT intervention with digital remote support was partly carried out during the COVID-19 pandemic. Through the experience of COVID-19 precautions, everyone learned more about digital communication, which also facilitated the use of this program over time. Initially, both the therapists and families had many technical problems, such as connection issues and difficulties logging into the web platform. During and after the COVID-19 precautions, the general internet presence and web-meeting technology have increased digital competence in the general population, which has also been found in other studies [46]. Although we did not formally measure cost-effectiveness from a healthcare perspective, it is quite obvious that time was saved for both the therapists and families. Furthermore, digital meetings are often shorter, and the format makes it easier to stick to the planned activity and subject. We received several reports from families who appreciated the digital meetings and spoke of them as convenient. Nevertheless, a prospective RCT is necessary to confirm these promising results and to truly evaluate the cost-effectiveness of this approach.

## 5. Conclusions

Using telerehabilitation for the further adjustment of the Baby-mCIMT program with remote coaching/supervision in a clinical setting was as beneficial as using Baby-mCIMT with in-person coaching/supervision provided during home visits. Our previous study has shown that in-person Baby-mCIMT is feasible; now, we can also conclude that remote Baby-mCIMT seems feasible, as most participating parents felt that the training was easy to perform and that remote support from an occupational therapist was of great importance. The parents also appreciated the additional online information provided. Remote coaching/supervision saves time for both therapists and families.

## Figures and Tables

**Table 1:** Demographic data of infants at baseline.

	Children at High Risk of UCP		
	Remote Coaching Baby-mCIMT (*n* = 20)	In-Person Coaching Baby-mCIMT (*n* = 18)	Statistics
Gestational age, weeks, mean (SD) (min–max)	38 (3.2) (31–41)	34 (6.5) (23–41)	*p* < 0.001
Age (CA) at baseline, months, mean (SD) (min–max)	7 (2.8) (3–13)	6 (1.7) (4–9)	*p* = 0.227
Gender, male/female	12/8	8/10	*p* = 0.300
Preterm/term	4/16	8/10	*p* = 0.106
HAI units at start (0–100), mean (SD) (min–max)	51 (8) (39–65)	52 (8) (42–69)	*p* = 0.599
Affected hand, left/right *n* (%)	11 (55%)/9 (45%)	7 (39%)/11 (61%)	*p* = 0.481
Weeks of inclusion, mean (SD) (min–max)	25.5 (5.3) (19–36)	22.7 (3.4) (17–29)	*p* = 0.026
AIMS at baseline	Missing, *n* = 8	Missing, *n* = 3	
<5	8	3	
5–10	2	3	
10–25	1	6	
>25	1	3	
Diagnosis CP,1 year of age			
Unilateral CP	17	18	
Bilateral CP	1	0	
No diagnosis	2 *	0	
Neuroimaging **	Missing, *n* = 3	Missing, *n* = 3	
No sign of lesion	0	0	
Focal infarct	8	4	
WMDI	7	7	
Miscellaneous	2	3	
Malformation	0	1	

* No diagnosis was noted in the medical records, but the children demonstrated clear signs of UCP. ** Analysis was mainly performed using magnetic resonance imaging close to term or at term-equivalent age in preterm infants, occasionally later on.

**Table 2:** Median and interquartile range (IQR) of HAI units and affected-hand raw scores for the different time points of assessment for both the remote and in-person coaching Baby-mCIMT groups.

Outcome	Baseline	After Period 1	Before Period 2	After Period 2	Difference within Groups: Baseline–after Period 2 ^a^	Difference between Groups: Baseline–after Period 2 ^b^
HAI units (0–100)						
Remote coaching Baby-mCIMT	50 (44:57)	53 * (50:59)	59 (49:64)	60 (55:70)	*p* ≤ 0.001	*p* = 0.803
In-person coaching Baby-mCIMT	50 (48:60)	57 (50:65)	60 (50:69)	60 (51:72)	*p* = 0.001	
Affected-hand raw score (0–24)						
Remote coaching Baby-mCIMT	8 (5:13)	9 * (6:13)	11 (7:15)	11 (9:18)	*p* ≤ 0.001	*p* = 0.942
In-person coaching Baby-mCIMT	10 (6:13)	13 (8:15)	12 (6:17)	13 (7:17)	*p* = 0.005	

* One is missing, ^a^ Calculated using the Wilcoxon signed rank test, ^b^ Calculated using the Mann–Whitney U-test.

**Table 3:** Responses from caregivers in the remote coaching Baby-mCIMT group about their experience of participation in the telerehabilitation program (n = 15) and responses from the in-person coaching Baby-mCIMT group in the previous RCT (n = 18).

Questions	%	%	%	%
How did the training go?	Very easy	Easy	Difficult	Very difficult
Remote coaching Baby-mCIMT	6	73	13	7
In-person coaching Baby-mCIMT	6	59	36	0
How significant was the support provided by the occupational therapist via the video meeting/home visits?	Of great significance	Of some significance	Of less significance	It would have probably gone well anyway
Remote coaching Baby-mCIMT	93	0	7	0
In-person coaching Baby-mCIMT	75	25	0	0
How did you perceive that the training affected your child’s hand function?	Good effect	Some effect	Minor effect	No effect
Remote coaching Baby-mCIMT	73	20	0	7
In-person coaching Baby-mCIMT	59	41	0	0
Would you recommend the training to other parents in similar situations?	Absolutely	Sure, it is ok	With some doubt	No
Remote coaching Baby-mCIMT ***	93	0	7	0
In-person coaching Baby-mCIMT	75	25	0	0
How laborious/difficult did you find it to complete the training?	Very difficult	Quite difficult	Pretty easy	Usually easy
Remote Baby-mCIMT *	8	46	38	8
What did you think about the material that was on the web?	Very informative and inspiring	Fairly relevant to my family	Not helpful	Can’t remember reading it
Remote coaching Baby-mCIMT **	36	64	0	0

* missing data n = 2, ** missing data n = 4, *** missing data n = 1.

## Data Availability

The data presented in this study are available on request from the corresponding author. The data are not publicly available due to privacy and ethical restrictions.
